# The Role of Inflammasome Activation in Early HIV Infection

**DOI:** 10.1155/2021/1487287

**Published:** 2021-09-20

**Authors:** Cyril Jabea Ekabe, Njinju Asaba Clinton, Jules Kehbila, Ngangom Chouamo Franck

**Affiliations:** ^1^Grace Community Health and Development Association, P. O. Box, 15 Kumba, Southwest Region, Cameroon; ^2^Mbonge District Hospital, South West Region, Cameroon; ^3^Faculty of Health Sciences, University of Buea, Cameroon; ^4^Health and Empowerment Foundation, Cameroon; ^5^Wum District Hospital, Wum, Northwest Region, Cameroon

## Abstract

The inflammasome pathway is an important arm of the innate immune system that provides antiviral immunity against many viruses. The main pathways involved in virus infections include the NLRP3, IFI16, and AIM2 pathways. However, a succinct understanding of its role in HIV is not yet well elucidated. In this review, we showed that NLRP3 inflammasome activation plays a vital role in inhibiting HIV entry into target cells via the purinergic pathway; IFI16 detects intracellular HIV ssDNA, triggers interferon I and III production, and inhibits HIV transcription; and AIM2 binds to HIV dsDNA and triggers acute inflammation and pyroptosis. Remarkably, by understanding these mechanisms, new therapeutic strategies can be developed against the disease.

## 1. Introduction

HIV infection is a global pandemic affecting about 38 million people worldwide, with approximately two-thirds of the patients found in Africa [1]. The global morbidity, mortality, and economic burden of this disease are quite remarkable. Furthermore, approximately 0.7% of people between 15 and 49 years are affected by this disease, signifying the burden of the disease in the working-age group [1]. The global impact of HIV infection has prompted more in-depth studies on molecular mechanisms that can be used as therapeutic strategies to improve the welfare of patients and prevent the spread and development of the disease. The HIV virus is an enveloped positive single-strand RNA (ssRNA) retrovirus that primarily infects CD4 T cells, as well as macrophages, and perhaps dendritic cells. It interacts with the CD4 receptors, and coreceptors (CXCR4, CCR5) expressed in these cells [2]. Moreover, HIV primarily interacts with the CCR5 coreceptors in infected cells. However, resulting variants from the high mutation rates of this virus are capable to bind the C-X-C chemokine receptor type 4 (CXCR4) coreceptor during the course of the disease in about 50% of the infected individuals, and their emergence is associated with a faster disease [3]. The high mutation rate of the virus is attributed to error-prone viral DNA synthesis and high recombination frequencies during reverse transcription. After binding of the virus to its target cells, the virus is internalized via clathrin-dependent endocytosis [4], which is mediated by glycoprotein gp21. Upon entry of the nucleocapsid into the cytoplasm, the virus undergoes partial uncoating exposing the viral genome and proteins to cytoplasmic sensors like DNA sensors, RNA sensors, endosomal TLRs (Toll-like receptors), and NLRs (nucleotide-binding domain, leucine-rich repeat-containing protein) receptors which can trigger the elimination of the virus. The NLRs are intracellular sensors vital in antiviral innate immunity. Inflammasomes are cytosolic multiprotein complexes critical for the activation of inflammatory caspases, required for activation of pro-IL1*β* and IL18, and characterised by acute inflammation and cell death [5]. NLRs are composed of three separate domains. The C-terminal region made of variable numbers of leucine-rich repeats (LRRs) that are thought to autoinhibit NLR in the resting state. The central nucleotide-binding and oligomerization (NACHT) domain is important for ATP-dependent oligomerization following inflammasome activation. The N-terminal domain comprising either a pyrin (PYD) or caspase activation and recruitment domain (CARD), necessary for protein-protein interactions. However, the N-terminal of NAIP family contains Baculovirus inhibitor-of-apoptosis repeats (BIRs) that distinguish it from other NLRs [6]. Inflammasomes are generally categorized into two: proinflammatory inflammasomes (NLRP3 and NLRC4) and anti-inflammatory inflammasomes (NLRP12, NLRX1, NLRC3, and NLRC5) [7–9]. These inflammasomes can either contain the PYD domain (NLRP3, NLRP1, AIM2, IFI16, and pyrin) or CARD domain (NLRC4, NLRC5) or the BIR domain (NAIP). The activation of the inflammasome pathway in response to various DAMPS (damaged associated molecular patterns) or PAMPS (pathogen-associated molecular patterns) occurs via two pathways: the canonical or noncanonical pathway. The classical canonical pathway is activated by various PAMPs (bacteria cell wall components, flagellin, bacteria toxins, and viral genomes) and DAMPS (ATP, organic and inorganic crystals, and reactive oxygen species) sensed by various NLRs. This leads to activation of caspase 1 which triggers the release of proinflammatory cytokines (IL1*β* and IL18), and Gasdermin-D, resulting in acute inflammation and cell death (pyroptosis), respectively [5]. The noncanonical pathway is activated in response to cytosolic lipopolysaccharides (LPS) derived from Gram-negative bacteria. The LPS binds to procaspase 11 in mouse, and procaspase 4/5 in humans leading to the activation of caspase 11 and 4/5, respectively. The activation of these caspases triggers Gasdermin-D-mediated pyroptosis [10]. The various forms of cell death associated with inflammasome activation include programmed lytic (pyroptosis and necroptosis) and programmed nonlytic (apoptosis) cell death. Studies have revealed that NLRP3, AIM2, and IFI16 sensors are pivotal for inflammasome activation in viral infections [11, 12]. Although inflammasome is activated in viral infection, it remains doubtful whether the response is helpful or detrimental to the host. Hence, this review is aimed at discussing the main inflammasome pathways important in HIV infection, and whether they can be harnessed as therapeutic strategies for the disease.

## 2. NLRP3 Activation in HIV Infection

**Figure 1:**
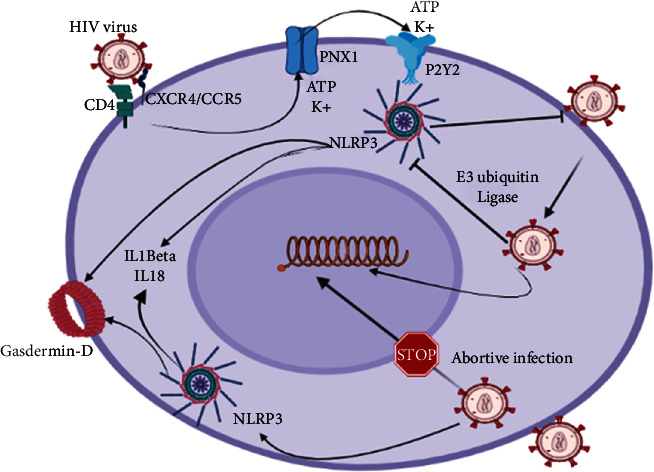
An illustration of the NLRP3 activation in HIV infection, and how HIV evades the NLRP3 inflammasome pathway. HIV infects target cells by interacting with CD4 receptors and coreceptors CXCR4/CCR5, which leads to the activation of the purinergic pathway via an increase in extracellular ATP and potassium ion [K+] efflux. Interaction between the activated purinergic receptor (P2Y2) and NLRP3 leads to activation of NLRP3 inflammasome which triggers acute inflammation, pyroptosis (via Gasdermin D), and inhibition of HIV entry. However, HIV evades the NLRP3 inflammasome pathway by activating the release of E3 ubiquitin ligase which ubiquitinates NLRP3 leading to proteasomal degradation. Also, abortive HIV infection of resting CD4 T cells triggers NLRP3-mediated pyroptosis which accounts for majority of CD4 T cell depletion and sustained inflammation.

The NLRP3 inflammasome pathway activation in HIV infection is postulated to result from multiple mechanisms. This includes ion flux like potassium efflux, mitochondria released of oxidative radicals, and lysosomal damage [13]. The binding of HIV-1 envelope glycoproteins to CD4 and coreceptors (CXCR4/CCR5) is associated with the activation of pannexin-1 hemichannels (PNX1) which leads to increased extracellular ATP and potassium efflux leading to the activation of the purinergic receptors (P2Y2) as seen in Figure 1 [14]. The activation of P2Y2 plays an important role in the pathogenesis of early HIV infection. Studies have shown that P2Y2 receptors interact with NLRP3 directly in the virological synapse formed between infected cells and uninfected target cells resulting in NLRP3 activation [15]. This interaction plays an important role in the regulation of viral entry into target cells and has been shown to increase rapidly with infection [15]. Additionally, there is more evidence supporting increased NLRP3 inflammasome activation in chronic HIV infection, which as an outcome triggers more inflammation and bystander damage of tissues [16]. This has been attributed to the effects of additional proteins like Tat and Vpr proteins in lymphocytes, microglial cells, and macrophages capable of stimulating further NLRP3 inflammasome activation [17, 18]. Therefore, targeting the molecular mechanisms associated with this interaction can be potential postulates for HIV therapy or vaccine discovery. Also, emerging evidence has revealed that the P2Y2 is paramount in HIV1 viral entry into target cells. It has been shown that P2Y2 enhances plasma membrane depolarization via activation of PYK2 which favours early fusion of the HIV1 membrane with that of target cells [15]. Developing evidence postulates that F actin polymerization is a key factor for enhancing the fusion of HIV 1 membrane with target cells. However, the role of F actin polymerization is independently modulated by the NLRP3 inflammasome. This is supported by evidence of enhanced F actin remodelling seen in NLRP3 depleted cells [15]. Thus, underscoring the role of NLRP3-mediated inhibition of cytoskeletal remodelling required for virus entry and subsequent accumulation of intracellular HIV1 capsid proteins. Despite the protective roles of the NLRP3 inflammasome pathway in inhibiting viral entry and intracellular accumulation of virus nucleocapsid, the HIV-1 virus has developed tenacious mechanisms for evasion. Studies have demonstrated that through posttranscriptional mechanisms like ubiquitination, HIV 1 is able to degrade NLRP3 [15]. The main mechanism encompasses the activation of E3 ubiquitin ligase and subsequent proteasomal degradation of NLRP3 (see Figure 1) [15]. Recently, the P2X7 receptor has been shown to play an important role in HIV-infected macrophages. Macrophages especially those of the CNS are resistant to virus-induced cytopathic effect and hence favour the survival of HIV virions and formation of virus containing compartments (VCC) [19]. These VCCs act as reservoirs that render the HIV virus poorly accessible to antiretroviral drugs and anti-HIV antibodies and also enhance virus spread through Trojan horse effect. NLRP3 activation in acute infection triggers accumulation of extracellular ATP (eATP) that activates P2X7 receptors in macrophages. The activation of P2X7 has been shown to remarkably contribute to the extrusion of VCC sequestered virions in infected macrophages without causing cell death [19]. Hence, the eATP/P2X7 pathway can be exploited pharmacologically to improve access to HIV reservoirs in cells which are not accessible to drugs or antibodies. Likewise, studies have also shown the importance of purinergic receptors in promoting HIV infection and chronic inflammation. A study carried on ex vivo human tonsil histoculture HIV infection model using different purinergic receptor antagonists revealed that P2X1 and P2X7 act independently as inhibitors of both HIV infection and HIV-induced inflammation [20]. Further analyses highlighted a remarkable decrease in HIV infection and production of IL-10 and IL1*β* following blockade of P2X1 and P2X7 receptors. From these findings, it was inferred that the use of drugs that block the above purinergic receptors can be utilized as new therapeutic strategies in the management of HIV-associated chronic inflammation and prevention of HIV infection. However, further research is needed in this subject to elucidate the underlying molecular mechanisms. Other factors including genetic polymorphisms like genetic variations of NLRP3 genes (rs10754558) and IL1B (rs16944, rs1143634) also protect against HIV [21]. However, variants in the IL18 promoter (rs1946518) increase the chances of HIV infection [22]. Likewise, studies reveal that bystander CD4 T pyroptosis account for about 95% depletion of CD4 T cells in HIV infection [23]. Further investigation revealed that NLRP3 mediated pyroptosis in response to reactive oxygen radicals in resting lymphoid CD4 T cells following abortive HIV infection accounts for bystander CD4 depletion. The high production of IL1*β* in abortive HIV infection via NLRP3-mediated caspase 1 activation remarkably contributes to sustained inflammation and disease progression [23]. This sustained inflammation is important in the clinical manifestation of cardiovascular diseases [24], neurological [25], and other inflammatory diseases in HIV infection. Recently, it was shown that HIV Tat proteins trigger NLRP3 and NLRC5 inflammasome activation in microglia cells surging the production of proinflammatory cytokines that contribute to sustained neuroinflammation and bystander damage [25]. Furthermore, HIV infects and triggers bystander damage of astrocytes and neurons in the brain via the ROS pathway and virus-induced mitochondrial damage [26]. The overall neuroinflammation contributes to neuroinflammaging responsible for some of the neurodegenerative diseases associated with HIV infection [25]. Thus, by studying the P2Y2-NLRP3 interaction in early HIV infection, molecular mechanisms underlying NLRP3-mediated bystander CD4 T cell depletion, and HIV neuroinflammation new approaches for the development of HIV vaccine or therapeutic interventions can be well exploited to improve the outcome of the disease.

## 3. IFI16 Activation in HIV Infection

**Figure 2:**
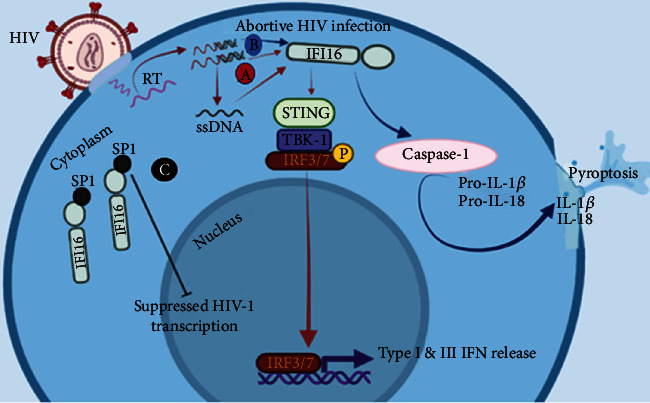
An illustration of the various mechanisms and pathways triggered by activated IFI16 in an HIV infection. (A) The activation of the STINK-TBK1-IRF3/7 pathway by IFI16 after the sensing of retroviral DNA intermediates or ssDNA. (B) The activation of the IFI16 inflammasome and resulting cell death due to the accumulation of incomplete DNA reverse transcripts of HIV during an abortive HIV infection. (C) Restriction of HIV-1 replication by IFI16 independently of immune sensing through the binding and inhibition of the host transcription factor Sp1.

The entire replication cycle of some lentiviruses including HIV involves a complex switching of different genetic materials as a result of the reverse transcriptase enzyme (RT). The different genomic switching involves a series of stages through which the genomic information is carried in the form of single-stranded (ss) RNAs, a hybrid of RNA/DNA, ssDNA, and dsDNA [27]. Microbial nucleic acids are known to be potent triggers of the innate immune response through their active recognition by some cytoplasmic or endosomal sensors (pattern recognition receptors (PRRs)) expressed by cells of the innate immune system. The recognition of viral nucleic acid structures by the innate immune system triggers an effector mechanism which usually results in the induction of interferons (IFNs) and IFN-stimulated genes (ISGs) [28–33]. IFNs can act as autocrine or paracrine factors on cells expressing the receptor. They are very essential cytokines necessary for the induction of an early defense against viral infections [34]. As described earlier, in order to initiate cellular infection, HIV-1 uses its envelope glycoproteins to engage the host cell CD4 receptor which causes a conformational change at the level of the envelop thereby enhancing subsequent binding to the host cell coreceptors (CXCR4/CCR5) to mediate cell entry [3]. Once inside the cell, the ssRNA must be converted to dsDNA [35]. This can only be accomplished with the help of the RT enzyme. The RT enzyme synthesizes a DNA strand from the ssRNA strand which forms a hybrid or duplex of RNA-DNA [35]. Subsequently, ssDNA is generated from which dsDNA is formed for host cell integration [35]. This complex genomic switch requires efficient recognition by different PRRs expressed by cells of the innate immune system in order to generate antiviral responses. It should be noted that the PRRs detecting dsDNA and the corresponding immune signaling cascades have been identified. However, sensors for ssDNA coupled with the appropriate downstream signaling cascades have not been reported. Reports have shown that the interferon-gamma-inducible protein 16 (IFI16) can serve as an immune sensor for retroviral DNA intermediates including the ssDNA [36]. IFI16 is a PYHIN protein (N-terminal pyrin and C-terminal HIN domain-containing) that binds DNA through their HIN200 domain (C-terminal DNA-binding region) [36, 37]. It has been demonstrated that the IFI16 can directly bind to stem-rich DNA structures of HIV-1 and induce the expression of type I (IFN-*β*) and III (IFN-*λ*) interferons in primary human macrophages [38]. The direct binding of IFI16 to the stem-rich DNA structures of HIV-1 resulted in the activation of the stimulator of IFN genes (STING) which then activates and recruits TANK-binding kinase-1 (TBK1), leading to the phosphorylation of the interferon regulatory factors (IRF) 3/7 which are important transcription factors for IFN-related genes (see Figure 2) [38].

Also, primary human macrophages deprived of IFI16 (IFI16 knockdown) displayed an amplified early viral replication and accelerated virus-induced cytopathic effects after HIV-1 infection [38]. This provides evidence on the role of IFI16 as a sensor for lentiviral RT products and control of the HIV-1 infection in macrophages through the induction of ISGs. In addition, reports have shown that the activation of the IFI16 inflammasome during an abortive HIV infection is linked to the depletion of CD4+ T cells through the process of pyroptosis mediated by activated caspase 1 [39]. This further suggests that IFI16 can sense incomplete DNA reverse transcripts of HIV that accumulate in abortively infected cells. The accumulation leads to caspase 1 activation, and subsequent death of these cells through the process of pyroptosis (see Figure 2) [39].

Reports have also shown that IFI16 can restrict HIV-1 replication independently of immune sensing by inhibiting the host transcription factor Sp1 (specificity protein-1) which is known to drive viral gene expression [40]. IFI16 is capable of binding to the Sp1 through the N-terminal pyrin domain following nuclear localization. The binding and inhibition of Sp1 by the IFI16 suppresses reactivation of latent HIV-1 proviruses in CD4+ T cells (see Figure 2) [40]. Therefore, an in-depth understanding of the immunobiology of the IFI16 and its protective role in HIV infection will be imperative for the development of an effective anti-HIV vaccine.

## 4. AIM2 Activation in HIV Infection

**Figure 3:**
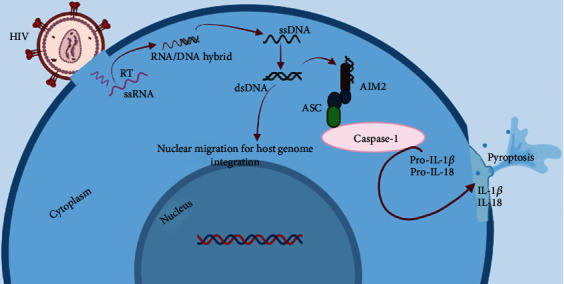
Illustration of the AIM2 inflammasome pathway activation during an HIV infection. The figure summarizes the stepwise genomic switching of the HIV upon cell entry. The AIM2 recognizes the viral dsDNA and interacts with the ASC. Activated ASC further recruits and activate the caspase 1 enzyme which triggers the process of pyroptosis (cell death).

AIM2 (absent in melanoma 2) is a cytoplasmic sensor of dsDNA which localizes to the cytosol. Like the IFI16, it is a PYHIN protein consisting of an N-terminal pyrin domain and a C-terminal HIN200 domain (binds dsDNA) [41, 42]. The activation of the AIM2 inflammasome has been reported in different viral infections including the HIV. Reports have shown increasing evidence on the upregulation of AIM2 genes in HIV-infected macrophages [43]. This highlights the possible involvement of the AIM2-mediated inflammasome during an HIV infection in nonpermissive cells. Moreover, it has been reported that there was an observed increase in the levels of the proinflammatory cytokine, IL-1*β*, through the AIM2 inflammasome activation in HIV progressors [16]. However, there is no report concerning the exact stage on the HIV infection cycle mediating the activation of the AIM2 inflammasome pathway. Generally, the activation of the AIM2 inflammasome has been hypothesized to be mediated through the interaction of the HIN200 C-terminal domain of AIM2 with the dsDNA (see Figure 3) [42]. The resulting dsDNA and protein heteroduplex through the pyrin domain (of AIM2) recruits the apoptosis-associated speck-like protein containing a CARD (ASC). This leads to the recruitment and activation of procaspase 1 to active caspase 1 (see Figure 3) [43]. Caspase 1 is known to cleave and activate the pyroptotic cytokines (IL-1*β* and IL-18) [43]. Since the HIV infection cycle involves the synthesis of dsDNA prior to host cell integration, it will be pivotal to further explore the possible involvement of the dsDNA sensor, AIM2. This can unravel the exact molecular mechanisms associated with the activation of the AIM2 inflammasome during HIV infection and provide a gateway for the creation of novel therapeutic avenues.

## 5. Conclusion and Future Perspective

HIV is an important pandemic and hence requires new and effective therapeutic and preventive interventions. From the above discussions, we can suggest that the inflammasome pathway plays an important role in the prevention of HIV entry, replication, and elimination of infected cells, as summarised in the supplementary file (available here). However, more studies are required to define the precise mechanisms in the inflammasome pathways to effectively eliminate or prevent disease progression. Hence, we recommend that further studies should be done on the role of inflammasomes in HIV infection. Through this, more therapeutic and preventive strategies can be utilised in the quest for the holy grail for this pandemic.

## Abbreviations

TLRs: Toll-like receptors
NLRs: Nucleotide-binding domain, leucine-rich repeat-containing protein
PNX1: Pannexin-1
IL: Interleukin
DAMPS: Damaged associated molecular patterns
RT: Reverse transcriptase enzyme
HIV: Human immunodeficiency virus
IFI16: Interferon-g inducible protein 16
STING: Stimulator of IFN genes
TBK1: TANK-binding kinase-1
IRF: Interferon regulatory factors
AIM2: Absent in melanoma 2
ASC: Apoptosis-associated speck-like protein containing a CARD
CARD: Caspase activation and recruitment domain
PYD: Pyrin domain
ISGs: Interferon-stimulating genes
IFNs: Interferons.

## Supplementary Materials

Supplementary Materials A summary of the role of inflammasome in HIV infection. In this figure, we illustrate the roles of NLRP3, IFI16, and AIM2 inflammasome pathways in HIV infection. Furthermore, we elucidate that NLRP3 inhibits HIV entry and triggers pyroptosis, IFI16 blocks viral transcription, induces pyroptosis, and increases interferon type I/III, while AIM2 also triggers pyroptosis in HIV infection.
