# Supplementary material for: The Role of Inflammasome Activation in Early HIV Infection
Source: J Immunol Res. 2021 Sep 20;2021:1487287. doi: 10.1155/2021/1487287 (PMC8478595; doi:10.1155/2021/1487287)
Supplement: Supplementary Materials — A summary of the role of inflammasome in HIV infection. In this figure, we illustrate the roles of NLRP3, IFI16, and AIM2 inflammasome pathways in HIV infection. Furthermore, we elucidate that NLRP3 inhibits HIV entry and triggers pyroptosis, IFI16 blocks viral transcription, induces pyroptosis, and increases interferon type I/III, while AIM2 also triggers pyroptosis in HIV infection. [file 1487287.f1.docx]

**Supplementary file**

**
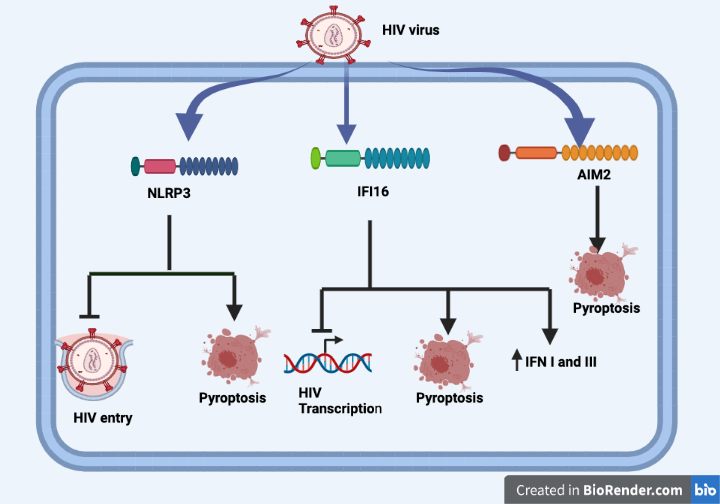
**

A summary of the role of inflammasome in HIV infection. In this figure we illustrate the roles of NLRP3, IFI16, and AIM2 inflammasome pathways in HIV infection. Furthermore, we elucidate that NLRP3 inhibits HIV entry and triggers pyroptosis, IFI16 blocks viral transcription, induces pyroptosis and increase Interferon type I/III, while AIM2 also triggers pyroptosis in HIV infection.
